# AGITATION IN ALZHEIMER'S DISEASE: A DECISION TREE FOR HEALTHCARE PROVIDERS

**DOI:** 10.1093/geroni/igac059.2013

**Published:** 2022-12-20

**Authors:** George Grossberg, Angela Sanford, Susan Scanland, Karen Tracy, Richard G Stefanacci

**Affiliations:** St Louis University School of Medicine, St Louis, Missouri, United States; St Louis University, St Louis, Missouri, United States; U of Pittsburgh, Pittsburgh, Pennsylvania, United States; GSA, Washington, District of Columbia, United States; Thomas Jefferson University, Philadelphia, Pennsylvania, United States

## Abstract

Agitation in Alzheimer's Disease (AAD) impacts nearly 80% of persons with Alzheimer's Disease and is a cause of significant distress for patients and family/professional caregivers.Clinicians need guidance relative to assessment as well as non-pharmacologic and pharmacologic treatment approaches to AAD. To this end, the Gerontological Society of America (GSA) convened a group of experts representing Geriatric Psychiatry, Gerontological Nursing, Geriatric Medicine, and Long-Term Care to develop a "Decision Tree for Healthcare Providers" in the evaluation and management of AAD. The "Decision Tree" is a practical, user-friendly template for clinicians which provides a step-by-step approach to differential diagnosis of AAD, including conditions such as delirium, pain/discomfort, environmental and psycho-social stressors. The "Decision Tree" also walks the clinician through a range of non-pharmacologic treatment options such as music therapy; activities therapy, environmental modification and de-prescribing. The "Decision Tree" highlights rational use of pharmacotherapies as well as their hazards in this vulnerable population.

